# Individual, social and environmental factors influencing physical activity levels and behaviours of multiethnic socio-economically disadvantaged urban mothers in Canada: A mixed methods approach

**DOI:** 10.1186/1479-5868-9-42

**Published:** 2012-04-13

**Authors:** Elizabeth D Mansfield, Natacha Ducharme, Kristine G Koski

**Affiliations:** 1 School of Dietetics and Human Nutrition, McGill University, Ste. Anne de Bellevue, Quebec, Canada; 2 Sandy Hill Community Health Centre, 221 Nelson Street, Ottawa, Ontario, Canada

**Keywords:** Women, Physical activity, Multiethnic, Socio-economic status

## Abstract

**Background:**

Existing data provide little insight into the physical activity context of multiethnic socio-economically disadvantaged mothers in Canada. Our primary objectives were: (1) to use focus group methodology to develop tools to identify the individual, social, and environmental factors influencing utilitarian and leisure time physical activities (LTPA) of multiethnic SED mothers; and (2) to use a women specific physical activity survey tool to assess psychosocial barriers and supports and to quantify individual physical activity (PA) levels of multi-ethnic SED mothers in Canada.

**Methods:**

Qualitative focus group sessions were conducted in West, Central and Eastern Canada with multiethnic SED mothers (n = 6 focus groups; n = 42 SED mothers) and with health and recreation professionals (HRPs) (n = 5 focus groups; n = 25 HRPs) involved in community PA programming for multiethnic SED mothers. Administration of the women specific Kaiser Physical Activity Survey (KPAS) tool was completed by consenting SED mothers (n = 59).

**Results:**

More than half of SED mothers were employed and had higher total PA scores with occupation included than unemployed mothers. However, nearly 60% of both groups were overweight or obese. Barriers to LTPA included the lack of available, affordable and accessible LTPA programs that responded to cultural and social needs. Concerns for safety, nonsupportive cultural and social norms and the winter climate were identified as key barriers to both utilitarian and LTPA.

**Conclusions:**

Findings show that multiethnic SED mothers experience many barriers to utilitarian and LTPA opportunities within their communities. The varying LTPA levels among these multi-ethnic SED mothers and the occurrence of overweight and obesity suggests that current LTPA programs are likely insufficient to maintain healthy body weights.

## Background

Physical inactivity is common among Canadian women of varying ethnicities and immigrant status [1-4]. This sedentary lifestyle plays a significant role in the health status of those women who are further disadvantaged by low socio-economic status [5]. A 22 year Canadian cohort study recently identified women, and especially those disadvantaged with respect to income and education, as the likeliest to experience decreasing trajectories of leisure time physical activity over their lifetimes [6]. Additionally, there is also evidence that both motherhood [7,8] and socio-economic status [6,9] are strong predictors of physical inactivity.

In 1974 the Lalonde Report in Canada recognized physical activity (as a sub-domain of lifestyle) as one of the four major determinants of health [10]. Recent research summarizing twenty year trends in leisure time physical activity (LTPA) among Canadian adults has suggested that despite an increase in the proportion of active Canadian adults over the last 2 decades, there has been an increase in the prevalence of self-reported inactivity-related diseases [11]. It has been suggested that more physical activity research should include domain specific physical activities (e.g. occupational, daily living) [11] and how activity in these domains may influence PA behaviour of socio-economically disadvantaged (SED) women [7]. Implicit in these suggestions is the need to address the individual, social and environmental determinants of physical activity so as to reduce the social inequalities and disparities of access [12,13]. Both the Integrated Pan-Canadian Healthy Living Strategy [14] and the Women’s Health Surveillance Report of the Canadian Institute of Health Research [9] have detailed specific recommendations for increased research to understand and to address these determinants of physical activity for SED mothers who may have limited opportunities to be physically active.

Because existing data provide little insight into the physical activity contexts of mothers of varying ethnicities and immigrant status living under socio-economically disadvantaged (SED) conditions in Canada, the Canadian Association for the Advancement of Women and Sport and Physical Activity (CAAWS) undertook a two year project (2007 to 2009) with the objective of identifying the individual, social and environmental factors that influence physical activity levels and choices of urban multiethnic SED mothers. Focus group discussions were conducted with multiethnic SED mothers and female health and recreation professionals (HRPs) involved in community physical activity programming for multiethnic SED mothers. The intent was to identify the individual, social and environmental factors that influence both utilitarian (daily living activities, including occupation) and LTPAs of urban multiethnic SED mothers. A secondary objective was to understand the type and amount of physical activity of multiethnic SED mothers in each of these domains and to characterize their psychosocial determinants of LTPA (perceived barriers, social support and self-efficacy) using the women specific Kaiser Physical Activity Survey (KPAS) tool [15]. An outcome of this project was the development of tools to assist HRPs in the planning and development of LTPA programs for SED mothers within their respective communities.

## Methods

### Experimental approach and recruitment

A purposive sampling strategy was used to recruit HRPs and multiethnic SED mothers from across Canada to participate in focus groups and to complete several questionnaires about physical activity between March and August 2008. To identify participants, study researchers collaborated with Federal Government regional managers in the Canada Prenatal Nutrition Program [16] and the Community Action Program for Children [16] to identify socio-economically disadvantaged urban communities in three regions across Canada (West, Central, East). Regional managers from these agencies and local community partners identified female community health organizers/promoters to act as local site coordinators and to recruit SED mothers and HRPs involved in community physical activity programming for multiethnic SED mothers. Community partners represented the physical activity interests of multiethnic groups, including francophone immigrant and resource centers, francophone sports federations, Family Services Early Childhood Programs, Immigrant Services, Aboriginal Head Start Programs, and parks and recreation and regional health units. Ethics approval was obtained from McGill University.

### Inclusion and exclusion criteria

Both HRPs and mothers had to be able to read and comprehend the project pamphlet and consent form in either English or French and to be able to meet at a designated time and place for participation in either an English or French focus group (HRPs, mothers) or to complete the physical activity survey (mothers) in English or French or have a community partner volunteer translator do this with them together with the local site coordinator. In addition, participating mothers had to self-identify as socio-economically disadvantaged (SED) using the MacArthur Scale of Subjective Social Status [17], have at least one child ≤14 yrs of age still living at home, be urban dwelling and not currently limited because of an illness, injury or disability. Each mother was given the choice to participate in both or either the focus group or the physical activity survey session. No honorarium was paid to SED mothers or HRPs but transportation to and from their focus group session was covered, as was child care for the duration of the session. Food vouchers were given to each SED mother at the end of her focus group and/or physical activity survey session(s). To maintain confidentiality and privacy, each participant was assigned a numeric identifier and the term “socio-economically disadvantaged” was removed from all study documents so as not to stigmatize participating/potential participating mothers.

### Focus group activities

A total of 42 multiethnic SED mothers participated in 6 focus groups and 25 HRPs participated in 5 focus groups in 3 regions of Canada (west, central and east). Initial pilot tests with 3 SED mothers and 2 HRPs determined the time needed for each component and ensured that the predetermined focus group questions were appropriate for generating discussion on the individual, social and environmental factors influencing utilitarian and LTPA choices of multiethnic SED mothers within their respective communities. All questions were framed in the context of social cognitive theory constructs (behavioural, environmental, personal) which theorize that self-efficacy, attitudes towards physical activity, perceived barriers, and past behaviour all influence intention and shape physical activity behaviour [18]. Bilingual (French, English) moderators facilitated each of the focus group discussions, allowing for flexibility in the focus group questions for generation of new areas of inquiry related to physical activity and to revisit earlier topics. A co-researcher took notes and wrote key points on a board for participants to view. These were used for summarizing the main points at the end of the discussion and asking participants if there were any missing key ideas. Each focus group lasted approximately two hours and was digitally recorded with permission of the participants.

Each focus group with multiethnic SED mothers began with an icebreaker in which mothers brainstormed for all the words that described what physical activity meant to them. Each mother created a physical activity pictogram describing the utilitarian and LTPA and relative levels (low/medium/high) that contributed to her lifestyle from childhood to present. The facilitator used the pictograms to engage the mothers in a discussion on their observed changes in physical activity levels since childhood. Mothers were probed on their barriers and social supports of a physically active lifestyle throughout their lifespan and their perceptions of their required supports for a physically active lifestyle in the future.

Each focus group with HRPs began with three questions to ascertain their beliefs about the health and fitness benefits of physical activity. HRPs were asked for their perceptions regarding the types and relative levels (low/medium/high) of utilitarian and LTPAs that contribute to multiethnic SED mothers’ lifestyles. This was used to facilitate a discussion on HRPs’ perceptions regarding the individual, social and cultural, organizational, and community level barriers and supports that influence physical activity behaviours and choices of multiethnic SED mothers. HRPs rated their self-efficacy for promoting and positively influencing multiethnic SED mothers’ physical activity behaviours and choices. HRPs were also asked how health professionals, community members, and organizations could better promote opportunities for multiethnic SED mothers to participate in physical activities within their communities.

### Survey tools

#### MacArthur scale of subjective social status

All participating mothers were asked to self-identify their socio-economic status, within the context of the Canadian community, using the MacArthur Scale of Subjective Social Status [17]. In a simple pictorial format, it presents a “social ladder” and asks individuals to place an “X” on the rung on which they feel they stand. Individuals placing themselves at rung 5 or lower were considered to be socioeconomically disadvantaged (SED). Local site coordinators described the ladder to each potential participating mother as follows:

"“At the top of the ladder are the people who are the best off in Canada – those that have the most money, the most education, and the most respected jobs. At the bottom are the people who are the worst off – who have the least money, least education, and the least respected jobs or no job. The higher up you are on this ladder, the closer you are to the people at the very top; the lower you are, the closer you are to the people at the very bottom. Where would you place yourself on this ladder?”"

#### Assessment of physical activity and psychosocial determinants of LTPA

The interviewer-led Kaiser Physical Activity Survey (KPAS) tool [15] was used to understand how the experience of physical activity in the context of everyday life has influenced the type and amount of physical activity of multiethnic SED mothers and to assess the psychosocial determinants of LTPA (perceived barriers, social support and self-efficacy). The KPAS tool has been validated in multiethnic populations of women with varying physical activity habits [7] and has demonstrated good reliability and accuracy to detect specific, habitual, daily activities such as housework/care giving, occupation and sports or exercise activities, the general level of physical activity involved in daily routines during the past year and personal feelings about exercise including perceived barriers, social support and self-efficacy [15]. The survey contains 75 items and takes approximately 30 minutes to complete in English or French (and up to an hour for those women who required a community partner volunteer oral translator for other languages). Timing for completion of the KPAS tool was entirely dependent on the availability of the SED mothers.

A detailed description of the KPAS tool and scoring procedures are described in detail elsewhere [7]. Briefly, the first four sections of the KPAS tool allow classification of physical activity status. Categorical responses, ranging from 1 for “never” to 5 for “always” regarding frequency of participation in sports/exercise and active living domains and ranging from 1 for “none” to 5 for “more than 30 hours/week” of employment created three semi-continuous activity indices (sport/exercise, active living, occupational). For the household/care giving section a 4 level categorical response ranging from 1 for “none” to 4 for “more than 20 hours per week” reflected the weekly time spent in care giving activities. For the sports/exercise section, an activity score describing the energy cost of physical activities was calculated from the mode, frequency, and duration of reported organized and recreational sports/exercise activities. These were converted into a LTPA score expressed as MET hours (kcal/kg per hour) and summed up over all reported sports/exercise activities. MET values calculated from the KPAS tool were then categorized into 3 levels of LTPA used in most surveillance studies (expressed in kcal/kg/day (KKD)): physically inactive < 1.5 KKD; moderately physically active 1.5-3.0 KKD; and physically active ≥ 3.0 KKD) [11]. Two total activity scores were created: Total Activity Score I (sum of all activity indices except occupation for all mothers) and Total Activity Score II (sum of all activity indices including occupational index for employed mothers only). The psychosocial section of the KPAS tool grouped responses into perceived barriers (external obstacles, heath constraints, lack of motivation, and time constraints), social support and self-efficacy.

#### Assessment of other covariates

In addition to completing the KPAS tool, mothers were also asked to self-report their current age, weight and height. BMI was calculated using self-reported body weight by height squared (meters^2^). BMI was further categorized into normal weight (< 25.0 kg/.m^2^), overweight (25–29.9 kg/.m^2^) and obese (≥30 kg/.m^2^). Ethnicity of mothers was based on the profile of the community group they were recruited from (i.e. Aboriginal Centre, African Immigrant Centre, Francophone Resource Centre, etc.).

#### Evaluation of focus group data

All focus group sessions were summarized using the notes and the verbatim transcribed digital recordings of the sessions. Ethnograph text analysis software (Ethnograph 6.0.1.0, Qualis Research, Colorado, USA) was used to organize the data. A codebook of common themes according to social cognitive theory constructs (behavioural, environmental, and personal) was created by the two facilitators and a framework organized through iterative reading of the transcripts. Sub-themes were determined using this framework and the text coded to identify patterns within the themes that showed a high degree of inter-rater agreement before key findings were established. Participants’ words were used to emphasize the key themes and sub themes.

Barriers and supports to utilitarian and LTPA were classified into three categories to represent the themes- individual, social and environmental - and summarized with examples, using explanatory quotations from focus group participants. Utilitarian physical activities are those activities of daily living, including active transport (e.g. walking or biking to work, school, shopping) and physical activity accumulated on the job and while performing household chores and childcare. LTPA included both organized and unstructured physical activities. Organized physical activities were considered planned and typically occured within a specific community setting. This included participating in a sports night (e.g. volleyball), taking an activity class (e.g. aqua fitness) or following a formal exercise program (e.g. strength and conditioning). Unstructured LTPA was performed outside of a formal, organized or structured setting. Examples included swimming, playing sports with children and traditional dancing with family and friends.

### Statistical analysis

Statistical analyses were performed using SAS for Windows (version 9.2; SAS Institute, Inc., NC). The KPAS tool domain specific activity indices were characterized by means, medians and 25th-75th percentiles. Distributions of demographic variables (age, BMI, leisure time physical activity levels) were characterized by means and standard deviations. The distributions of the four semi-continuous activity indices (sport/exercise, active living, occupational, household/care giving) were tested for normality using the Shapiro-Wilk test. T-tests for comparison of means contrasted sports/exercise levels amongst employed and unemployed mothers. Chi-square tests were used to assess for differences in the proportions of perceived barriers, social support and self-efficacy of employed versus unemployed SED mothers. A level of P < 0.05 was considered statistically significant. A scatter plot of BMI and kcal/kg/day (KKD) was included using cut points, as defined previously, for leisure time physical activity levels (sedentary, active and moderately active) [11].

## Results

### Quantitative methods - Kaiser physical activity survey (KPAS) tool

**Figure 1:**
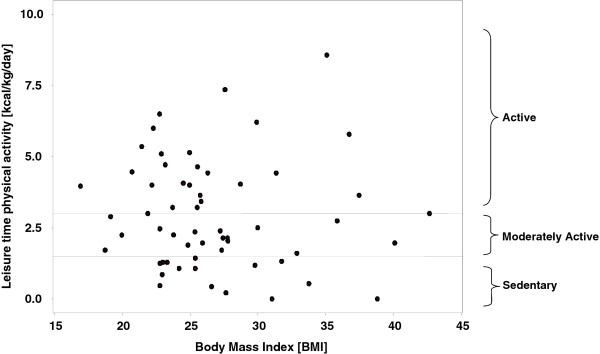
Leisure time physical activity level and BMI of SED mothers.

Quantitative assessment of physical activity levels of 59 multiethnic SED mothers was assessed with the KPAS tool. All 59 mothers completed the 1^st^ 4 sections but only 52 of the SED mothers completed all 5 sections, due to personal time constraints. More than half of all mothers surveyed were employed. More than half of both unemployed and employed SED mothers were overweight or obese but there were no significant differences in age, BMI, or ethno-cultural grouping between the unemployed and employed SED mothers (Table 1). Variability was greatest for the occupational index (interquartile range 2.1) and smallest for the household/care giving index (interquartile range 0.9). There were no differences in the total activity scores (Total Activity Score I) between employed and unemployed SED mothers when occupational index was excluded however inclusion of the occupational index significantly increased employed mothers total activity level (Total Activity Score II) (p < 0.0001). Only two SED mothers reported no sports/exercise in the past year. The most popular activity was walking (41%) followed by aerobics (19%) and swimming (8%) (data not presented). The mean energy expenditure in sports/exercise was highly variable and this did not differ by employment status (Table 1). There was no apparent relationship between SED mothers’ self-reported leisure time sports/exercise energy expenditure and BMI (Figure 1).

**Table 1:** Personal characteristics and physical activity levels reported by SED mothers completing the KPAS tool

	**Total**	**Unemployed**	**Employed**	P-value*
Number of mothers (n)	59	22	37	
Age, years [(mean, SD)]	35.7 (7.7)	35.8 (7.1)	35.6 (8.1)	0.93
Body Mass Index, kg/m^2^ [n; (%)]				
Normal weight, BMI < 25	25 (42.4%)	9 (40.9%)	16 (43.2%)	0.90
Overweight, BMI 25 – 29.9	22 (37.3%)	9 (40.9%)	13 (35.1%)	
Obese, BMI ≥30	12 (20.3%)	4 (18.2%)	8 (21.6%)	
Ethno-cultural grouping [% (n)]				
Multi-ethno cultural Anglophone	19 (32%)	11 (50%)	8 (22%)	0.27
Multi-ethno cultural Francophone	23 (39%)	4 (19%)	19 (51%)	
Aboriginal	17 (29%)	7 (32%)	10 (27%)	
Kaiser Physical Activity Survey Indices [median (25^th^-75^th^ percentiles)]				
Sports/exercise index ^a^	3.0 (2.0 − 3.8)	3.0 (2.0 − 3.8)	3.0 (2.3 − 3.8)	0.66
Household/Care giving index ^b^	3.0 (2.6 − 3.3)	3.1 (2.5 − 3.4)	2.9 (2.6 − 3.2)	0.55
Active living index ^c^	3.0 (2.5 − 3.5)	3.0 (2.8 − 3.3)	3.0 (2.5 − 3.5)	0.45
Occupational index ^d^	2.4 (1.0 − 3.1)	1.0 (1.0 − 1.0)	2.9 (2.5 − 3.5)	<0.0001
Total Activity Score (TAS) I ^e^ [mean (SD)]	8.8 (1.9)	8.9 (1.9)	8.8 (1.8)	0.89
Total Activity Score (TAS) II ^f^ [mean (SD)]	N/A	N/A	11.7 (2.2)	N/A
Leisure time activity levels [mean (SD)] ^g^				
Kcal/kg body weight/day (KKD)	3.0 (1.9)	3.0 (1.7)	2.9 (2.1)	0.91
Kcal/day (KD)	209.9 (152.1)	214.2 (138.8)	207.3 (161.5)	0.87
Kcal/week (KW)	1469 (1065)	1499 (971)	1451 (1131)	0.87

^*^P value for significance of differences between TAS I (employed) vs. TAS II (employed).

^a^ Calculated as the average of 5-level (range 1–5; low – high) categorical responses to 3 universal questions about participation in sports/exercise plus a 5-level ordinal mapping of a continuous score derived from frequency and duration of participation in three most frequent sports/exercise activities.

^b^ Calculated as the average of 5-level categorical responses to 11 questions about household/care giving activities. ^c^ Calculated as the average of 5-level categorical responses to 4 questions about active and sedentary living habits.

^d^ Calculated as the average of 5-level categorical responses to 8 questions about occupational activities plus 3 level ordinal mapping based on Labour Department coding of occupational titles. The value of “1” represents “None” with respect to employment.

^e^ Sum of all indices, except occupational index, for all mothers.

^f^ Sum of all indices, including occupational index, for employed mothers only.

^g^ Calculated from the mode, frequency, duration of specific sports/exercise activities converted into a leisure time physical activity score expressed as MET hours per week, then multiplying frequency by duration and by MET value and summed up over all activities. One MET (metabolic equivalent) is approximately 1 kcal/kg per hour and is equivalent to the oxygen consumption required at rest.

**Figure 2:**
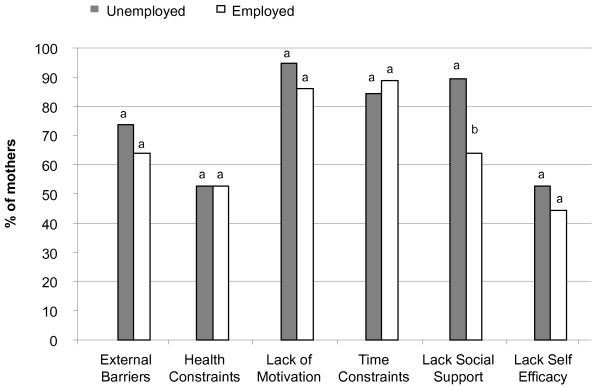
**Psychosocial correlates of physical activity of socioeconomically disadvantaged mothers.** Bars with different letters indicate a significant difference between% of unemployed and employed mothers *p* < 0.05.

Psychosocial determinants of leisure time physical activities (organized and recreational sports/exercise) of SED mothers derived from the KPAS tool were not significantly different between unemployed and employed mothers (Table 2). Key factors preventing most of the mothers from getting the amount of exercise they felt they wanted or needed were time constraints and a lack of childcare, a lack of intrinsic motivation (self-discipline), interest or enjoyment, a lack of energy, and a lack of sport skills or knowledge (Table 2). The majority of SED mothers indicated that they lacked the social supports for getting the amount of exercise they wanted or needed. When responses were grouped into the 6 overall categories of external barriers (4 categories), social support (1 category) and self-efficacy (1 category) there were no significant differences in any of the psychosocial determinants of exercise behaviour between employed and unemployed mothers with the exception of lack of social support which was significantly higher for unemployed mothers (Figure 2).

**Table 2:** Psychosocial determinants of LTPA behaviour of SED mothers

		Total	Unemployed (UE)	Employed (E)	*P*-value
		**N = 59**	**N = 22**	**N = 37**	
**PERCEIVED BARRIERS % of mothers for whom the following prevents them from getting the amount of exercise they want or need sometimes/often/always:**					
*External Obstacles*	No exercise/sport equipment	45.5	52.7	41.6	0.44
	Lack of skills or knowledge	52.7	63.2	47.1	0.26
	Lack of facilities or space	40.0	36.9	41.7	0.73
*Mental & physical health constraints*	Self-conscious about how I look when I exercise	40.0	36.9	41.7	0.73
	Feelings that I don’t deserve the time to exercise	16.4	15.8	16.7	0.93
	Concern for personal safety	29.1	15.8	36.1	0.11
	Lack of energy	69.1	79	63.0	0.25
	Lack of good health	9.1	0	13.9	0.15
	Fear of injuring myself	38.2	47.4	33.4	0.33
*Lack of motivation*	No one to exercise with	45.5	52.6	41.7	0.44
	Lack of good weather	45.5	52.6	41.7	0.44
	Lack of interest or enjoyment	60.0	52.6	63.9	0.42
	Lack of self-discipline	72.7	79	69.4	0.45
*Time constraints*	Lack of time	78.2	73.7	80.6	0.56
	Lack of childcare	60.0	73.7	52.7	0.13
**SOCIAL SUPPORT**	**% of mothers for whom at least 2–3 times a month or more, friends or family usually…**				
	exercised with them	21.8	15.8	25	0.43
	offered to exercise with them	16.4	5.3	22.2	0.11
	gave them encouragement to exercise	30.9	15.8	38.9	0.04
**SELF EFFICACY**	**% of mothers who maybe can/ are probably sure they can/sure they can…**				
	exercise when they are feeling sad or highly stressed	56.4	42.2	63.9	0.12
	exercise when work, family or social life take a lot of your time	38.2	26.3	44.4	0.19
	set aside time for regular exercise	60.0	57.9	61.1	0.82

### Qualitative methods - focus groups with SED mothers

Just over one third (n = 22) of the SED mothers who completed the KPAS also chose to participate in a focus group discussion. A total of 35 SED mothers participated in one of 6 focus groups in 3 regions across Canada. Participant profiles of SED mothers reflected the ethnicity of the communities from within which they were recruited (Table 1). In each focus group SED mothers discussed what “physical activity and exercise” meant to them, their personal levels and types of physical activities over their lifespan, their barriers to physical activity, and the supports that have enabled them to be physically active.

#### Lifetime trends in physical activity

Personal pictograms captured each mother’s trends in utilitarian and LTPA throughout her lifespan. These consistently highlighted that LTPA was highest throughout the secondary school years. Utilitarian physical activity began in adolescence when young girls began to share the responsibility for household tasks including care of their siblings. This and occupational activity (starting with their first jobs) were the most important contributors to daily physical activity levels as girls transitioned into young adulthood. Life changes that occurred with motherhood affected young women’s physical activity behaviours, decreasing leisure time sports and exercise and increasing household/childcare and activities of daily living. Motherhood was supportive of leisure time physical activities only in those mothers who played with their school aged children.

"“*Like if you have kids, you have to teach them, they study at home, they need more activity. Playing together, riding bicycles, walking, dancing, yoga, swimming, shopping, walking the malls, traveling.”*"

For many mothers however, motherhood resulted in minimal LTPA with their children.

"*“Suppose I try to do the dancing with music, with my kids, I have two years-old daughter, it is very hard to do the yoga thing with her because she doesn’t like yoga -it’s just one-on-one because our children, she’s just four years-old so she can’t play soccer or something like this.”*"

Promoting overall health was the most salient personal factor that influenced SED mothers’ participation in LTPA. Self-identified health benefits included feeling good, getting fit and having fun, better energy levels, stress reduction, improved self-esteem, weight loss and healthy aging. Mothers stressed the important role of physical activity for weight loss, in particular the loss of that weight gained during pregnancy:

"*“When I was school-age or teenager, I never thought about losing weight, when I got pregnant I gained lots of weight, now I think oh my god how can I lose the weight? That’s the thing working all the time in my mind. So I do physical activity more, like walking”*"

"*“Exercise, whatever you can get, I’m waiting until the weather change so I can walk more. Eating, uh, healthy eating, it’s true, but I don’t watch my diet. They go together, but most, to me, I don’t watch what I eat but I like to go out there and do exercise to lose the weight.”*"

#### Individual barriers

SED mothers expressed a wide range of barriers to LTPA including a lack of physical skills to be able to participate in sports/exercise, body image issues related to being overweight, a lack of priority for physical activity, as well as guilt for actually taking the time to be physically active (Table 3). Across all focus groups the most common intrapersonal barriers to both utilitarian and LTPA included a lack of motivation and fatigue. Difficulties finding appropriate clothing as an overweight mother (sport bra, swim suits) limited many mothers’ participation in sports and other physical activities.

**Table 3:** Major themes and sub-themes of barriers to and supports of physical activity with representative quotations arising from focus group discussions with multiethnic SED mothers

**Individual factors**	
**Barriers**	**Lack of energy and motivation** *“…All your energy (goes) to the kids, only them.”* *“…free time from kids; we can read something, read some books, listen to music or watch movies…”*
	**Body image** *“Once I became a teenager I had cramps every time…because I didn’t want to mess up my hair, I didn’t want to change in front of the girls.”* *“Here I come and I’m just in big sweater and pants and then I don’t feel comfortable next time I go to the group right because I feel like I stick out.”*
	**Physical skills** *“some of us don’t know how to swim so we let the kids go in the pool and then watch while they’re swimming.”*
	**Priority** *“Someone has to take initiative so we can get together and discuss how to improve our daily life”*
	**Guilt** “*.I tell the kids I’m going to lose weight (by doing exercise), I never lose weight; they (children) told you, already you are mom, why you going to lose weight? You’re not becoming a good mom anymore; you just think about yourself, you’re selfish.”*
**Supports**	**Health benefits** *“to lose weight is a motivator” (to be physically active)…* *“Walking is a practical sport because we can do lots with it.”* *“Physical activity helps me to sleep better”* *“physical activity helps me….physically and mentally to be happy…”* “R*ight now I’m a diabetic, my sugars are very high, I’m trying to get my sugars down and my husband is trying to get me back to the gym…”*
**Social factors**	
**Barriers**	**Family expectations** “*Yeah, back home we always provide, as a mom, give, give, give. Expectations is too high, expectations as a mom is very high, so that’s how they expect, so if you say I want to go to school, I want to exercise, oh my God there is an issue.”* *“In our culture, when you become a mom, your life is ended”.*
	**Weight-related teasing** *“As the mom ok sometimes you feel, ok your mom is fat, because of you the kids may bully, your mom is chubby, look at your mom. There was a rap song saying: look at your mother, she’s fat..the child is six, seven, eight years old, he can get defensive about when somebody sees it’s his mom, like for example I have a seven year-old who always says mommy don’t come to the school because some of my friends says that you are fat.”*
	**Culture/religion** “…*I know back home all my brothers know how to bike, I never learned. Even if we wear pants we don’t even know how to ride bikes, because this was wrong”* *“In my country, when a girl like me wanted to do sports and she did it, one man said: since when did you lose your ‘Indian-ness’?”*
	**Lack of financial resources** *“. some people they don’t have a bus pass; they can’t afford to buy it. If your budget is low, how’re you going to do that?”* *“Clothing, yeah clothing, it’s expensive; because we don’t wear the swimming suits…”*
	**Lack of spousal support** *“Husbands don’t know that kind of information that we need sometimes help, mental help, sometimes I feel I have two kids, oh my god I need some time for me, at least thirty minutes, I want to do something for me”* *“Like my husband, he does anything he wants, he has plenty of time after work, he has groups where he exercise, whereas me I’m stuck with the kids all the time. No obligations for the man to look after the kids. So that we do need a lot of support as a mom and the children.”*
**Supports**	**Family activities** *“Like if you have kids, you have to teach them, they study at home, they need more activity. Playing together, riding bicycles, walking, dancing, yoga, swimming; shopping, walking the malls, traveling.”* *“It’s something to do with our kids, to do it together, like with the baseball with the kids, it’s more fun”*
	**Traditional Activities** “*Like traditional dancing, I’d like to learn, I’ve got my daughter, I’d like to teach her, and I’ve got me, get myself going again.”*
	**Family support** *“I go to my parents’ cottage. The kids are at the beach while I do other activities.”*
	**Friends’ support** “*If you have friends that support you, that want to do it with you, that helps you to do those physical activities”*
**Environmental factors**	
**Barriers**	**Financial Costs** *“…when we want to register our kids or even me and my husband, a family you know, it’s too expensive to go to the community centre…”*
	**Stigma with low-income subsidies** *“There are some but you have to be below the poverty level to have access.”*
	**Lack of transportation** *“It depends also sometimes transportation; you need bus…if it’s so far. Yeah, too far or not on a bus route, or four buses/too many buses to get there”*
	**Unsafe environments** “. *even with the community around here like, the areas are so, you don’t trust to walk at night…a lot of the places I wouldn’t go out by myself, down here it’s just kind of - you don’t know”*
	**Poor climate** *“The summer, soccer you can do but the winter you stay at home”* *“.sometimes, especially in storm (winter), I stay 2,3,4 days at home, I can’t go outside….”,*
	**Lack of Multicultural Resources** *“the problem is that I do not speak English, only French and this makes communication very difficult..”*
	**Lack of Childcare** *“Because you can participate more when you know that you have child care”* *“… they used to have child care and then had exercise on the site and they stopped that and that was a big loss for us so most of the mothers stopped going there.”*
**Supports**	**Professional Support** *“For me, you need a program, a person that gives you support but support with experience, professional support”*
	**Availability of subsidies** “The last minute club, its good, if nobody registers for that particular course then we get it for free…” *“I know YMCA have good subsidy for low income people.”*
	**Presence of Cultural Community Centres** *“Everybody; they make you feel welcome, you don’t get that sense of clique, intruder feeling when you come in and you’re new; everybody’s welcome; no judgment.”* *“I need the socialization; when we do things here, we play baseball, we go swimming, we do all this stuff …”*
	**Outreach and Networking** *“And then if I come across other native people, the single moms, or they don’t even have to be single, I’ll say oh my god, down at the centre we have the blah blah blah and that’s where word of mouth comes in.”* *“A sense of community, trust…our nativeness…a sense of security… when I say my band, no one asks me what instrument I play…”*

Interpersonal barriers included nonsupportive cultural norms for both utilitarian and LTPA (e.g. cultural acceptance for women to ride a bike either as a mode of transportation or for sport/exercise) and being intimidated by the social environment through negative experiences about body size and shape including weight-related teasing of children of overweight SED mothers (Table 3). A lack of spousal support was a consistent barrier to LTPA, as were family obligations and expectations. Financial barriers to LTPA were consistently mentioned in all focus groups. This included transportation and clothing costs.

#### Social barriers

Community barriers (program and childcare costs) rendered physical activity opportunities unaffordable to many SED mothers. The lack of culturally appropriate sport and exercise programming for women, a lack of women coaches/leaders, unavailability of recreational facilities (including limited or inconvenient hours of operation for those SED mothers who work shift work), a lack of childcare, and a lack of physical activity resources in languages other than English were also reported. Web-based information was often not updated with currently available recreation and childcare opportunities and was usually available in English only, despite the multilingual characteristics of the population these promotional materials were to serve.

Policy barriers included limited accessibility to subsidies such as recreational facility access cards to those whose family income was below a set amount that defined them as “poor”. However the social stigma of proving low income prevented some of these mothers from acquiring these subsidies. Furthermore, some mothers indicated that although their family income was above the cut-off criteria they nevertheless had insufficient financial resources to participate in leisure time programs because childcare costs were still too high for those mothers that had more than one child. These mothers talked about the potential utility of the social status ladder tool that was used to recruit them into this study as preferred accessibility criteria for subsidized programs (Table 3).

#### Environmental barriers

The location of the recreational facility was a barrier when it was not served by public transportation or was too far removed from the community that it was designed to serve (e.g. numerous bus transfers) (Table 3). Across all focus groups, accessibility to a safe and secure environment was a key barrier to both utilitarian and LTPA (Table 3). SED mothers identified concern for safety as an environmental barrier to active transport (e.g. walking through unlit areas at night). Mothers were concerned about crime and the lack of safe neighbourhoods and playgrounds for participating in LTPA. Mothers felt that the outdoor parks in downtown neighbourhoods were rundown, unsafe or inaccessible with a stroller. Dirty needles, cigarettes, crack pipes could also be found, further limiting their accessibility for mothers with young children looking for safe places to play together. Cold temperatures and the lack of snow removal further limited access to the parks throughout the winter and was a noted key barrier to utilitarian physical activity. Furthermore, the height of snow banks at intersections was a challenge for mothers walking to and from the bus and grocery stores with children in strollers. Aboriginal and immigrant multicultural mothers indicated that the decrease in utilitarian physical activity in the winter resulted in weight gain, leading to further health conditions including diabetes (Table 3).

### Supporting factors for physical activity

#### Individual supports

Perceived health benefits, in particular maintenance of a healthy weight and/or weight loss, were motivators for these women to be physically active in their leisure time and to incorporate daily physical activities such as walking. A support system of family and friends was essential for those SED mothers to consistently maintain a physically active lifestyle that included LTPA. Traditional activities (e.g. traditional dance) were considered an important physical activity for aboriginal mothers and their children to do together. Many mothers identified the influence of “champions” for social networking, outreach and social support (e.g. health advice, information on programs and services, cultural acceptance, sharing child care responsibilities), and information on evaluating physical health as important. These champions took the form of health and recreational professionals as well as peers who led by example (Table 3).

#### Social and environmental supports

SED mothers indicated that there should be an increase in the availability of subsidies and bursaries for SED mothers to be able to take part in LTPA at recreation centres with childcare provided simultaneously. Mothers also suggested that there should be an increase in the number of hours of affordable childcare within their respective communities. Subsidies, when accessible, supported SED mothers in their pursuit of a more physically active lifestyle in their leisure time. Native and multicultural community recreation centres were identified as secure and trusted environments where physical activities, including traditional activities, could be done in a trusted environment. Outreach and networking were important for delivery of structured physical activity programming information within aboriginal and multicultural communities. In addition, the warmer summer weather was identified by many mothers as an environmental support of both utilitarian and LTPA.

### Qualitative methods - focus groups with health and recreation professionals (HRPs)

Five focus groups were conducted with a minimum of three female HRPs of varying backgrounds and levels of experience in 3 regions of Canada (Table 4) In general, HRPs were confident advising SED mothers about the health and fitness benefits of physical activity. The HRPs were also confident in their abilities to assist SED mothers in overcoming barriers to LTPA and establishing a regular leisure time physical activity program. They also considered themselves to be effective promoters of SED mothers’ participation in physical activities (Table 5). The health benefits of physical activity for SED mothers were considered a priority by HRPs. They agreed that 30 minutes of brisk walking most days of the week would suffice for improving health. However, they also considered moderate to vigorous exercise of longer duration as essential for improving health (Table 5).

**Table 4:** Focus Group Characteristics of HRP’s

**Focus Group**	**Region/Location**	**Language of Focus Group**	**# HRP Participants**	**HRP Profile based on community contact**
**SED Mothers**	West/Calgary	French	5	African
				Quebecois
		English	12	African
				Asian
				Caribbean
	East/Halifax	English	7	Aboriginal
	Central/Ottawa	English	8	African
		French	7	African
				Caribbean
				Franco-Ontarian
		English	3	Caucasian
**Health & Recreation Professionals**	West/Alberta	French	4	Program managers Social workers
		English	4	Multicultural program supervisor
				Program manager
				Health promoter Registered Dietitian
	East/Halifax	English	3	Aboriginal community social workers
	Central/Ottawa	French	8	Recreation program coordinators
				Program manager
				Health promoters
				Social worker
				Nurse
		English	6	Recreation manager
				Nurses
				Health promoters

**Table 5:** Physical activity advice: practices and beliefs of health and recreational professionals (HRPs) dealing with multiethnic socio-economically disadvantaged (SED) mothers in three regions of Canada

**Below are some statements about advising multi ethnic SED mothers about physical activity and exercise for their general health and well-being. For each statement, please mark your level of agreement.**	**%** **Strongly agree**	**%** **Agree**	**%** **Neutral**	**%** **Disagree**	**% Strongly disagree**
Whenever I am dealing with a SED mother, I always promote the general benefits of physical activity.	26	39	26	9	0
I feel confident to advise SED mothers about the levels of physical activity recommended for health gain.	48	52	0	0	0
Aside from exercise prescribed for treatment purposes, I feel confident that I can help SED mothers set goals and develop a plan to maintain a regular physical activity program.	13	65	22	0	0
I can help SED mothers to overcome the barriers they have to establishing a regular physical activity program.	21	54	8	8	8
I don’t think that I can influence the level of participation in general physical activity among SED mothers.	0	25	8	38	29
**Below are some statements about physical activity. For each statement, please mark your level of agreement.**					
To improve your health it is essential to do moderate to vigorous exercise for at least 20 minutes, 3 times a week.	35	65	0	0	0
Exercise doesn’t have to be done all at once – blocks of 10 minutes are okay to improve your fitness level.	12	70	9	9	0
Half an hour of brisk walking on most days of the week is enough physical activity to improve your health.	61	30	0	9	0

Range of responses (n = 23–25 respondents).

### Perceived barriers of HRPs

#### Individual barriers

Generally, HRPs perceived that SED mothers accrued sufficient daily utilitarian physical activities but that few mothers had LTPA and/or regular exercise programs due to family demands and a lack of supports (i.e. childcare or a women’s support group).

"“I feel most of them have more activities, they are walking, they don’t have a car, or taking the stuff they have to get around to get services, the kind of stuff they do, they probably are on the move more.”"

"*“…coming to work on Wednesday morning, seeing a young mom pushing a double stroller with 2 kids in it in all that snow storm, the street and sidewalks were horrible, it probably took her10 minutes to walk up to the end of the street where she was heading and I thought that this is really physical activity.”*"

#### Social and environmental barriers

HRPs discussed the misconceptions and stereotypes surrounding “socio-economically disadvantaged” and the lack of physical activity program leaders who understood the needs of SED mothers who have cultural, religious and ethnic needs that are quite different from the societal norms (Table 6). Across all focus groups, HRPs indicated that there was insufficient funding for physical activity programming for SED mothers and their families, and a lack of partnership between health and recreation professionals to create physical activities that are coordinated, connected, responsive, effective and sustainable (Table 6). The public health nurses who developed and implemented physical activity strategies targeted to the SED population of women in their community expressed their outrage with the municipal recreation planners whose bottom line mandate is to offer programs that cover all their costs, effectively canceling out programs for those that cannot afford the registration fees. A municipal recreation planner indicated the need for a higher-level directive from Parks and Recreation with respect to programming for the SED community:

**Table 6:** HRPs’ perceptions of barriers and proposed solutions for increasing physical activity opportunities for SED mothers

**Perceived barriers of health and recreational professionals**	**Proposed solutions and examples**
Misconceptions and stereotype of who is socio-economically disadvantaged	Talk with SED mothers and assess (through women-specific physical activity survey tools) what are appropriate activities for their different cultural, age and life situations. Assess what they would like to do; what their barriers and limitations are; and how physical activity fits into their culture.
Not enough program leaders who understand SED mothers and their needs	Develop mentorship programs for participants in Women’s Only physical activity/exercise programs (e.g. Women Alive) to become an instructor and teach the program.
	Offer on-going support to the trainees.
	Invite SED mothers to participate in physical activity program development
Insufficient financial resources for physical activity programs for SED mothers	Generate more funding and foster a greater understanding from municipal parks and recreational groups that having SED mothers physical activity programs will profit the community by being more welcoming to everyone.
Lack of partnership between health and Recreation professionals	Individual organizations need to create partner networks to work together to create physical activities that are coordinated, connected, responsive, effective and sustainable. This requires collaboration and coordination between those who have the recreation facilities, and those who have the communication and capacity-building in the community.
	Public health can pay for instructors and supply the in-person support needed for the group.
	Offer programs at times when there is childcare available and a convenient time for the mothers rather than when the recreation center is not busy or it is not their most profitable time.
SED mothers feel intimidated by some leaders	Set the goal to have fun. Build a sense of belonging and security. Hire welcoming and non-judgmental leaders.
	Hire leaders who understand SED mothers i.e. having people leading who have had the experience in your own community will result in an immediate bond and better understanding of the immediate issues SED mothers face.
Not enough mental health workers	More mental health workers to help SED mothers overcome some of the mental health barriers post-partum mothers experience.
Lack of Francophone physical activity resources in Alberta	Integrate Francophone programs into recreation centres;
	Hire bilingual professionals and recruit bilingual volunteers to contribute to physical activity programs;
	Political request for bilingual services for all Francophone Canadians (not just immigrant Francophone) within the Francophone community in Alberta potentially using Centre Accueil Nouveaux Arrivants Francophones (CANAF)

"*“.somebody at the top needs to realize because I am driven by money, that my goal is not to make a huge profit, I can run a program but I at least need to recover the instructor’s cost.”*"

The cold weather environment throughout the winter months was also perceived to be a barrier to physical activity for those new immigrants coming from warmer countries.

### Proposed solutions of HRPs

Education of multiethnic SED mothers and their spouses about the importance of LTPA for women’s health, increased availability of culturally competent, bilingual and multilingual staff in francophone and multicultural resource centres, and improved partnerships between health and recreation professionals were considered integral components for increasing physical activity levels of SED mothers. Community assessments by HRPs could determine the physical activity needs of SED mothers, their barriers and limitations, and how to best integrate appropriate activities for different cultures, ages and life situations.

## Discussion

This study provided insight into the individual, social and environmental factors that influence both utilitarian and leisure time physical activity (LTPA) of multiethnic socioeconomically disadvantaged (SED) mothers in three urban regions of Canada. Using a mixed methods approach that included focus groups, assessment of psychosocial correlates of physical activity, and quantitative assessment of domain specific physical activity levels, our findings highlight, as others have [19-21], the importance of considering barriers and supports that influence both utilitarian and LTPA of SED mothers. Our major findings were as follows:

1. (Individual) Achieving a healthy body weight was an important individual factor influencing LTPA habits of SED mothers but as reported by others [7,22,23], domestic activities (household chores and childcare) were significant contributors to daily physical activity levels of SED mothers, leaving them with little time or energy for LTPA. These SED mothers considered LTPA to be an important tool for weight loss and/or maintenance of a healthy weight as others have previously suggested [24,25]. However, our findings suggested that these mothers were unable to maintain healthy weights with their reported levels of physical activity. A second contributing factor which has also been described by others [26,27] was the inability of these SED mothers to set aside time for regular LTPA when they are dealing with home, work or other life stressors.

2. (Social) The presence of a social support network including spouse, extended family, community champions and HRPs, was a major factor influencing LTPA behaviours of SED mothers which has been reported in the US [28]. Similar to other studies [29,30], structured physical activity programs and group activities organized by professionals and/or done in partnerships with other groups within their communities improved the self-efficacy of SED mothers to plan time for physical activity within their days and become more physically active.

3. (Environmental) A key environmental factor limiting physical activity levels and behaviours of these SED mothers was the lack of available, affordable, and accessible LTPA programs with childcare that fit into the mothers’ schedules, and that responded to their cultural and social needs. Finally, the summer climate promoted both utilitarian and LTPA whereas the winter climate was a major barrier to both.

SED mothers considered LTPA to be an important weight loss tool, similar to findings of the 2005 Canadian Community Health Survey, Cycle 3.1, which showed that those who were active in their leisure time were more likely to have lower levels of overweight and obesity [31]. More than half of SED mothers completing the KPAS tool were overweight or obese and there was no apparent relationship between LTPA energy expenditure and BMI, suggesting that LTPA programs were not meeting mothers’ needs for management of healthy weights. SED mothers’ reported mean LTPA energy expenditures of 3.0 (±1.9) kcal/kg/day (KKD), which is a threshold, of physical activity equivalent to walking for ≤ 60 minutes/day. Although currently considered as “active”, the recently recommended ≥ 60 minutes of *moderate* to vigorous intensity exercise per day [32] may be needed to manage body weight and attenuate age-related weight gain in multi-ethnic SED mothers. Thus LTPA programs may need to focus on increasing opportunities for year round moderate to vigorous physical activity for multi-ethnic SED mothers.

A sense of trust in the community where physical activity programs/efforts were offered was an important motivation for SED mothers to be physically active. Aboriginal women attending private fitness centres did not feel a sense of community trust and support. This made it somewhat uncomfortable and daunting for them to commit to a regular program of physical activity, despite significant health issues that they felt would be better controlled with regular LTPA. Aboriginal mothers felt that the existence of a native friendship centre with structured physical activities for women and their families improved their involvement in LTPA. Being together as a band brought a greater sense of security. The selection of culturally specific champions from among SED clientele with expertise in traditional dance was noted as an important motivator for increasing physical activity levels amongst the SED mothers. SED mothers also noted that LTPA programs involving these “champions” were important for social networking, social support, and for fostering the development of social relationships and community cohesion. As with other research in culturally diverse women [33,34], an absence or lack of women-only exercise and sport programs were described as important deterrents for mothers whose cultures or religions necessitated women only programs.

Environmental barriers limited the accessibility of SED mothers to LTPA opportunities within their own communities. HRPs identified the oppositional clash in values that pits the LTPA needs and interests of the SED mothers against the demands for profit making in the recreation sector. SED mothers indicated that LTPA program subsidies designed to include financially disadvantaged women and their families, further stigmatized these mothers with an onerous and invasive process of proving poverty in order to qualify. Furthermore, subsidies did not necessarily provide a discount on childcare, effectively excluding low income SED mothers from participating in programs when they could not bring their children. A recent report by the Canadian Fitness and Lifestyle Research Institute showed that while the percentage of municipalities who offer programming and scheduling to low income groups has increased dramatically in Canada, the number who offer fee discounts or subsidies for low income adults has remained unchanged [35].

This study was limited by its cross sectional design which meant that causality could not be attributed. There was also the possibility of selection bias as a result of the purposive sampling strategy used. The small sample size was driven by the small number of SED communities within Canada where HRPs were conducting targeted physical activity programming for SED mothers. A key strength of this study was the use of mixed methods to examine the barriers and supports for physical activity that SED mother’s experience that could inform future program development efforts.

### Implications

The 2010 Toronto Charter for Physical Activity made a call to action to create sustainable opportunities for physically active lifestyles for all Canadians [12]. To do this, organizational programmers and community policy makers must address the social, cultural and physical environment and policies promoting physical activity within their communities, as has been concluded by researchers in the US [36]. An integral component is the involvement of SED mothers of young children in the discussions and development of local physical activity promotion strategies, including increasing partner support, social advocacy, and capacity building, as this has already shown some success in Canada [22] and the US (27).

With the current funding environment it is imperative that HRPs make efficient use of existing resources to maximize participation of SED mothers in LTPA programming within their communities. As part of this project tools were developed to assist HRPs to assess SED mothers’ physical activity needs and to develop and plan for LTPA programs that address the issues of availability, affordability and accessibility of LTPA programs while creating positive physical opportunities for mothers to lead healthy lives for themselves and their children. These tools are available for download in French and English on the CAAWS website “Mothers in Motion” http://www.caaws.ca/mothersinmotion/e/lowstatus/tools.cfm

## Conclusions

This study identified lack of time, childcare, social and financial support as key barriers to LTPA of SED mothers. Concerns for safety, nonsupportive cultural and social norms and the environment (seasonal climate) were identified as barriers to both utilitarian and LTPA. The presence of social support networks, including spouse, extended family, community champions and/or HRPs were key supports for SED mothers to incorporate LTPA within their lives. The varying LTPA levels amongst these multi-ethnic SED mothers and the rates of overweight and obesity suggests that current LTPA programs are likely insufficient to maintain healthy body weights. Opportunities for year round moderate to vigorous physical activity programs that have the capacity to meet the social, cultural and health needs of multi-ethnic SED mothers and their families may be needed to manage and achieve healthy body weights. Integral to this is the involvement of SED mothers in the discussions and development of local physical activity promotion strategies. Furthermore, integration of culturally competent HRPs is an important component for this community development approach. Accessible, available and affordable LTPA opportunities for SED mothers that are culturally responsive give SED mothers’ the potential to achieve their self-identified health benefits of LTPA. In the future, with more communities having the tools to assess their communities for development of LTPA programming, intervention studies can be better designed to meet SED mother’s needs.

## Competing interests

The authors declare that they have no competing interests.

## Authors’ contributions

EM conceived of, designed and implemented the study and performed the ethnographic analysis of the focus groups (with ND), participated in the statistical analysis and interpretation of data, and drafted the manuscript. ND coordinated the recruitment for the study, assisted in the implementation of the study, assisted in the ethnographic analysis of the focus groups (with EM), and transformed the Kaiser Physical Activity Survey output into a database form. KK participated in the analysis and interpretation of data and helped to draft the manuscript. All authors read and approved the final manuscript.
